# Needle-Probe Optical Coherence Tomography for Real-Time Visualization of Veress Peritoneal Needle Placement in a Porcine Model: A New Safety Concept for Pneumoperitoneum Establishment in Laparoscopic Surgery

**DOI:** 10.3390/biomedicines10020485

**Published:** 2022-02-18

**Authors:** Eric Yi-Hsiu Huang, Meng-Chun Kao, Chien-Kun Ting, William J. S. Huang, Yi-Ting Yeh, Hui-Hsuan Ke, Wen-Chuan Kuo

**Affiliations:** 1 Department of Urology, Taipei Veterans General Hospital, Taipei 11217, Taiwan; yhhuang3@vghtpe.gov.tw (E.Y.-H.H.); jshuang@vghtpe.gov.tw (W.J.S.H.); 2 Department of Urology, School of Medicine, National Yang Ming Chiao Tung University, Taipei 11221, Taiwan; 3 Shu-Tien Urological Research Center, National Yang Ming Chiao Tung University, Taipei 11221, Taiwan; 4 Institute of Biophotonics, National Yang Ming Chiao Tung University, Taipei 11221, Taiwan; biophotonics@ym.edu.tw (M.-C.K.); ytyeh3@vghtpe.gov.tw (Y.-T.Y.); hhke@vghtpe.gov.tw (H.-H.K.); 5 Department of Anesthesiology, Taipei Veterans General Hospital, Taipei 11217, Taiwan; ckting@vghtpe.gov.tw

**Keywords:** Veress needle, laparoscopy, peritoneal cavity, optical coherence tomography

## Abstract

The safe establishment of pneumoperitoneum is a critical step in all laparoscopic surgeries. A closed pneumoperitoneum is usually obtained by inserting a Veress needle into the peritoneal cavity. However, there is no definite measure to visually confirm the position of the Veress needle tip inside the peritoneal cavity. This study aimed to describe a method of real-time visual detection of peritoneal placement of the Veress needle using an incorporated optical coherence tomography (OCT) probe in a porcine model. A 14-gauge Veress needle was incorporated with a miniature fiber probe to puncture the piglet’s abdominal wall into the peritoneal cavity. A total of 80 peritoneal punctures were attempted in four piglets. For each puncture, continuous two-dimensional OCT images of the abdominal wall were acquired for real-time visual detection of the needle placement into the peritoneal cavity. Characteristic OCT image patterns could be observed during the puncturing process, especially a deep V-shaped concave pattern before the peritoneum puncture, which was a crucial feature. A statistical difference in the OCT signal standard deviation value also indicated the differentiability of images between the peritoneum and extra-peritoneal tissue layers. A success rate of 97.5% could be achieved with the guidance of the OCT images. OCT images translate the blind closed technique of peritoneal access into a visualized procedure, thus improving peritoneal access safety.

## 1. Introduction

Laparoscopic surgery is a significant milestone in the contemporary history of surgery. It has the advantages of smaller surgical wounds, less blood loss, less postoperative pain, quicker recovery, and shorter hospital stay than its open counterpart. Thus, laparoscopic surgery has become the standard of care in many operations. The first and essential step for every laparoscopic surgery is gaining access to the peritoneal cavity and creating a pneumoperitoneum. After successfully establishing the pneumoperitoneum, the laparoscopic trocars can be safely inserted, and the remainder of the surgical procedure can be performed smoothly. Despite rapid advances in laparoscopic surgery in the last three decades, there remains no clear consensus regarding the optimal method of gaining access into the peritoneal cavity [1]. Closed pneumoperitoneum is usually obtained by blind insertion of a Veress needle into the peritoneal cavity, which is regarded as the gold-standard technique [2]. Nevertheless, the insertion of a Veress needle may carry potential risks such as bowel and vascular injury [2,3]. It has been reported that at least 50% of major complications occur before commencing the planned surgery [4,5]. A systematic review of 38 articles that included 696,502 laparoscopic procedures identified 1575 injuries caused by Veress needle insertion, with a complication rate of 0.23% [6]. Wherry et al. reported that the number of vascular injuries in laparoscopy was 2 per 10,000 procedures, and a serious complication associated with mortality occurred in 3.3 per 100,000 procedures [7]. Jamil et al. performed a comparative study of closed vs. open method of pneumoperitoneum and found that the rate of visceral injury due to Veress needle was as high as 1.17% [8]. In addition, preperitoneal insufflation and failed entry into the peritoneal cavity is another important issue regarding inadequate insertion depth of Veress needle. Mikhail et al. performed a randomized controlled study using a Veress needle insertion with and without concomitant CO_2_ insufflation. They found a comparable incidence of preperitoneal insufflation between the two groups (4.4% vs. 6.1%, *p* = 0.99) [9]. Therefore, confirming safe entry of the Veress needle into the peritoneal cavity is of utmost importance for successful laparoscopic surgery.

In children, especially young children, the introduction of a Veress needle is more difficult due to the smaller abdominal cavity and the extensibility of the peritoneum. Due to the above, the number of injuries is slightly higher than in adulthood [10], although numerous studies support the safe use of Veress needle in children [11,12].

Various tests have been used to confirm the position of the needle tip inside the peritoneal cavity, including the hissing sound test, hanging drop test, and many others [13]. However, the puncturing process of Veress needle placement and the confirmatory tests for the needle tip position are all performed in a blind fashion that requires subjective judgment and carries significant uncertainties and risks. The puncture risks may be lowered if needle penetration can be visualized or imaged during the puncturing process. A few reports describe optical methods to confirm entry of the Veress needle, but they are not widely used [14,15].

Swept-source optical coherence tomography (SSOCT) can provide high-resolution and real-time images with high tissue discriminative ability. It is clinically helpful in the field of ophthalmologic examination [16]. The SSOCT systems combined with a miniature fiber optic probe can be inserted into different luminal tissues to observe and detect tissue changes [17]. We recently examined the feasibility of using the needle probe SSOCT to identify the epidural space and demonstrated the high sensitivity and specificity of this method [18]. In this study, we conducted a preliminary experiment in a porcine model that aimed to combine fiber-probe SSOCT with a Veress needle for real-time visual detection of needle placement into the peritoneal cavity before the establishment of pneumoperitoneum.

## 2. Materials and Methods

### 2.1. Study Design

This study was approved by the Ethics Committee of Taipei Veterans General Hospital (Taipei VGH IACUC 2019-083; 4 July 2019). Four Chinese native pigs with an average weight of 25 kg were studied. Experimental animals were obtained from an outsourced specialized facility outside the laboratory where we conducted the experiments. All stages of our study were conducted on an anesthetized porcine model under isoflurane anesthesia to minimize animal suffering. A lethal dose of KCl (2 mEq/kg) was injected to euthanize the animals at the end of the procedure.

### 2.2. Incorporated Veress Needle and Fiber-Probe SSOCT System

**Figure 1:**
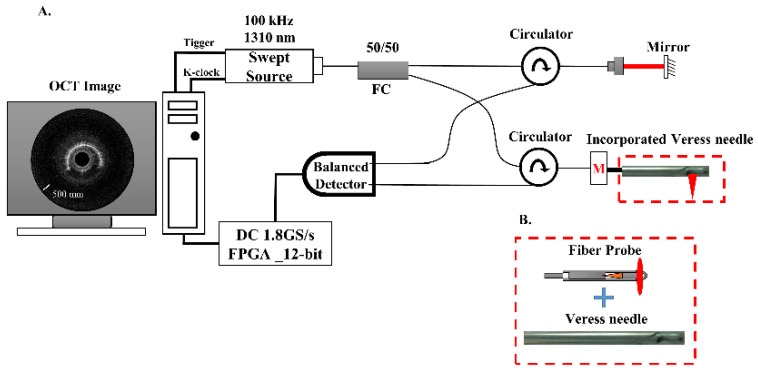
(**A**) Schematic layout of incorporated Veress needle-based swept-source optical coherence tomography (SSOCT) system. An OCT image of the needle’s inner layer obtained using the OCT needle probe system. FC: fiber coupler, DC: digitizer card, FPGA: field-programmable gate array. (**B**) Schematic of the Veress needle combined with the fiber probe.

Figure 1A shows the schematic of the SSOCT system. The swept-source has a central wavelength of 1310 nm and a bandwidth of 100 nm. The swept frequency is 100 kHz, and the output power was approximately 30 mW (Axsun, Billerica, MA, USA). Swept-source light was split into sample and reference arms by a 1 × 2 coupler. In the sample arm, the fiber probe (with a 0.9 mm outer diameter) incorporated with a 14-gauge Veress needle (ENDOPATH^®^ Pneumoneedle Insufflation Needle, 120 mm, Ethicon Endo-Surgery Inc., Cincinnati, OH, USA) (with a 1.6 mm inner diameter and 2.1 mm outer diameter) and a rotary motor was used to perform side-view imaging. Figure 1B shows a picture of the incorporated Veress needle in the sample arm. A balanced detector (PDB470C, Thorlabs, Newton, NJ, USA) was used in the detection arm, and 2000 A-scans were acquired to construct a two-dimensional (2D) OCT image, corresponding to an effective rotational rate of 50 rounds/s. The OCT signal was collected by 12 bit, 1.8 GS/s digitizer cards (ATS9360, Alazar Technologies, Pointe-Claire, QC, Canada). Accordingly, the SSOCT probe system has an axial resolution of approximately 17.5 μm in air (corresponding to 15 μm in tissue). OCT images were real-time displayed in 50 frames/s by field-programmable gate array (FPGA) processing.

**Figure 2:**
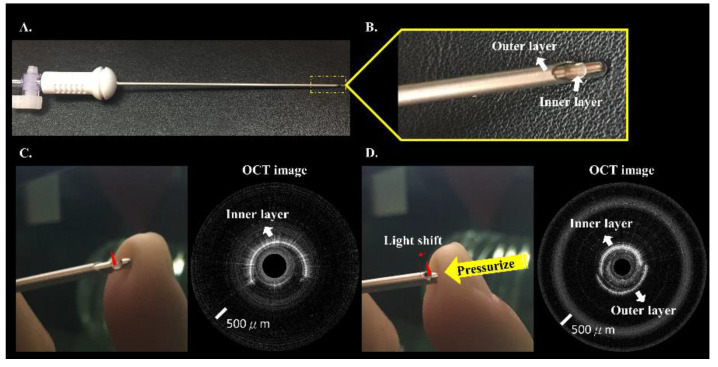
(**A**) Photograph of incorporated Veress needle. (**B**) Enlargement of the yellow area of A. (**C**) Light-spot position of incorporated Veress needle; OCT image of the needle’s inner layer obtained by the OCT system. (**D**) The needle inner and outer layers were simultaneously shown in an OCT image when the light-spot position was shifted due to outside pressure.

Figure 2 shows the Veress needle and the OCT image from the incorporated Veress needle. The Veress needle (Figure 2A,B) has a two-metal-layer design. OCT images were acquired by circumferentially scanning the optical fiber probe with a rotational motor. Semi-circle of the OCT image (Figure 2) showed strong reflections from the inner layer of the needle. When the needle tip was pressured, the remaining semi-circle of the OCT image scanned the needle’s outer layer, as shown in Figure 2D. The output light position depends on the tissue resistance feedback when the operator punctures the abdomen.

### 2.3. Quantitative Parameter Measurement and Statistical Analysis

The standard deviation (STD) of OCT signal value in each of the 2D OCT images was calculated to nonsubjectively discriminate the peritoneum from the extra-peritoneal tissue objectively. The linear mixed model (LMM) analysis was used to calculate the normality of the data distribution. In addition, LMM analysis with compound symmetric covariance structures for repeated measures was used to evaluate whether the STD in the OCT images (i.e., including 80 punctures) of the peritoneum differed from that of the extra-peritoneal. A *p* value of less than 0.05 was considered statistically significant. Receiver operating characteristic (ROC) curves were used to show the discrimination capability when the STD value was used to discriminate between the peritoneum layer and extra-peritoneal tissue. The area under the ROC curve (AUC) was used to measure the diagnostic discrimination of a test. All statistical analyses were performed using SPSS software version 24 (IBM Corp., Armonk, NY, USA).

### 2.4. Porcine Model

**Figure 3:**
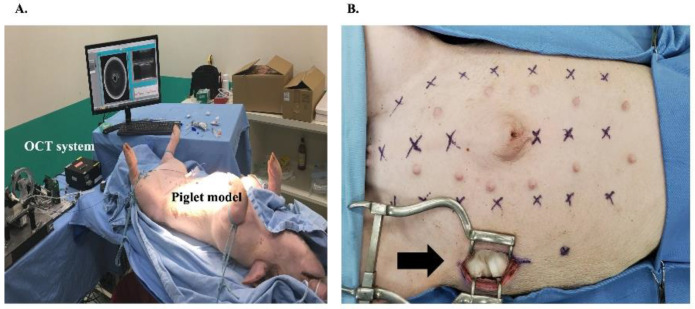
(**A**) Photograph of our SSOCT system with the Veress needle probe and piglet model. (**B**) The designated puncture sites on the piglet abdominal wall. A 5 cm full-thickness abdominal wall incision was created at the lateral abdominal wall to visualize needle placement after the puncture (arrow).

A porcine model was used because of the similarity of its abdominal wall anatomy to that of humans. Four laboratory piglets with an average weight of 25 kg were intubated and ventilated after general anesthesia with intramuscular tiletamine–zolazepam (5 mg/kg). Anesthesia was maintained using isoflurane. The piglets were placed in the supine position for Veress needle placement. The fiber SSOCT probe incorporated with a 14-gauge Veress needle was used to puncture the peritoneal cavity through the abdominal wall. Real-time OCT imaging was used to guide the Veress needle placement at different abdominal wall sites either in the midline or paramedian region, approximately 4 cm lateral to the midline of the abdominal wall (Figure 3A). A 5 cm full-thickness abdominal wall incision was made at the lateral abdominal wall to visualize needle placement after the puncture (Figure 3B).

### 2.5. Experimental Conditions

For each piglet, 6 midline and 14 paramedian abdominal wall punctures were designated in advance before puncturing the abdominal wall. The combined Veress needle and fiber-probe SSOCT system was utilized to puncture the abdominal wall into the peritoneum with real-time OCT image monitoring. Once the Veress needle was punctured into the peritoneal cavity, the Veress needle tip’s actual position was confirmed by observation from the lateral abdominal wall incision. The success rate of puncturing into the peritoneum was calculated.

### 2.6. Primary and Secondary Outcomes

The primary outcome of the study is the success rate of peritoneal puncture with the incorporated Veress needle. The secondary outcome is the discrimination capability between the peritoneum and extra-peritoneal tissue.

## 3. Results

**Figure 4:**
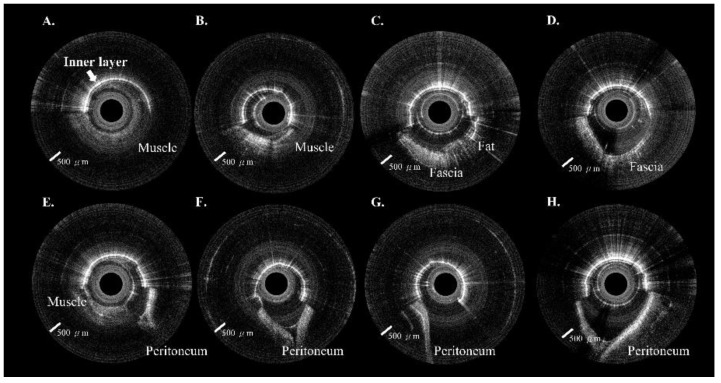
Veress needle probe punctured the midline of the abdomen. (**A**–**D**) Approximately half of the OCT image shows the extra-peritoneal tissue. (**E**–**H**) Approximately half of the OCT image shows the peritoneum.

**Figure 5:**
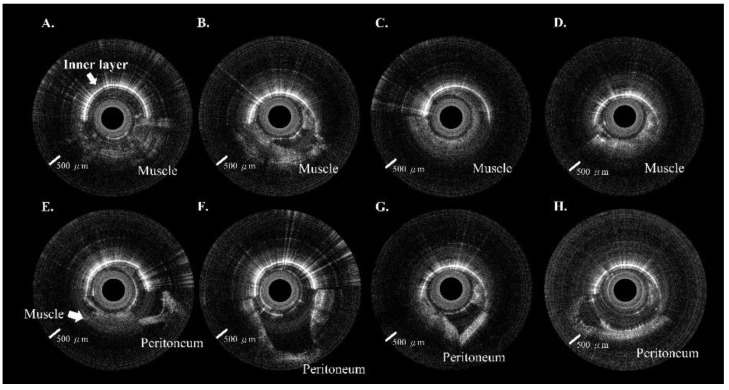
Veress needle probe punctured the paramedian region of the belly. (**A**–**D**) Around half of the OCT image shows the extra-peritoneal tissue. (**E**–**H**) Around half of the OCT image shows the peritoneum.

**Figure 6:**
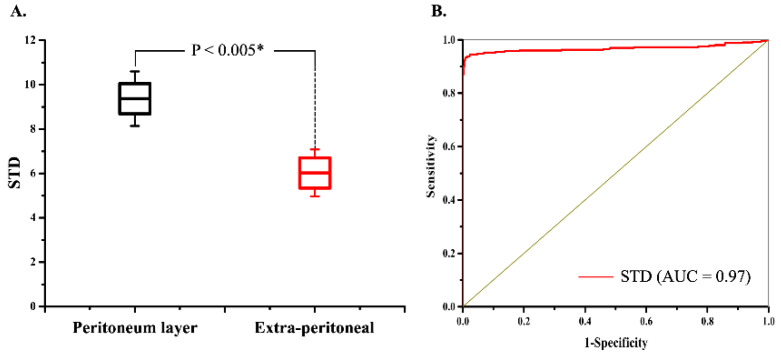
(**A**) Distributions of the OCT STD value from regions in the peritoneum and extra-peritoneal tissue. (**B**) Receiver operating characteristics (ROC) curves show the OCT STD value’s capacity for discrimination between the peritoneum and extra-peritoneal tissue. * *p <* 0.005.

Eighty peritoneal punctures were attempted in the four piglets; 78 (97.5%) were successful. No intra-abdominal organ injury occurred during the experiments. Each site included ten images from the extra-peritoneal tissue (i.e., muscle, fat, or fascia) through the peritoneum. A total of 800 in vivo 2D OCT images were obtained from these 80 puncture sites. The OCT images in Figure 4A–D and Figure 5A–D included muscle, fat, and fascia tissue obtained from extra-peritoneal tissue when the incorporated Veress needle punctured the abdominal midline (Figure 4) and paramedian (Figure 5). The strong scattering of light in Figure 4E–H and Figure 5E–H shows the OCT images of the peritoneum while the incorporated Veress needle was punctured through the peritoneum. The characteristic features of the different tissue layers are summarized in Table 1. A video showing the puncturing process with animation and OCT images are available in the supplementary material (Video S1). Although we found a similar V-shaped concave pattern in both the extra-peritoneal fascia and peritoneal OCT images, the OCT image intensity of the peritoneum was stronger and had low image penetration versus the extra-peritoneal fascia OCT image. A statistical difference in the STD value indicated the differentiability of images between the peritoneum layer (9.36 ± 1.22) and extra-peritoneal tissue (6.02 ± 1.05), as shown in Figure 6A. The AUC (Figure 6B) for the ROC curve indicated that the STD value had a high discriminatory capacity (AUC = 0.97) for discriminating between the peritoneum layer and extra-peritoneal tissue.

**Figure 7:**
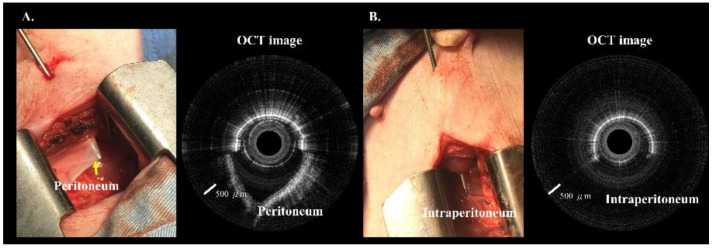
(**A**) Photograph of the incorporated Veress needle at the peritoneum. Around half of the OCT image show the peritoneum. (**B**) Photograph of the incorporated Veress needle punctured through the peritoneum. Around half of the OCT image show the intraperitoneal space.

**Figure 8:**
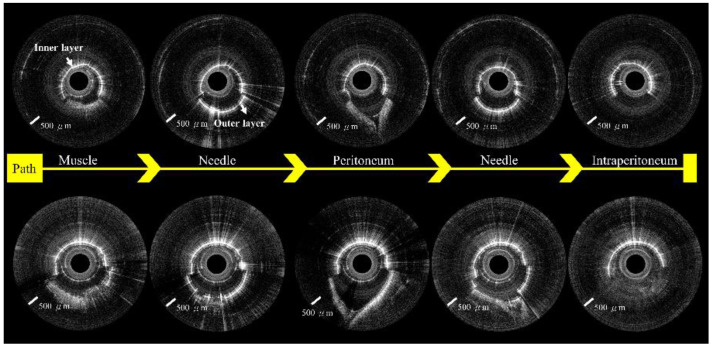
OCT images show the puncture path from the midline of the abdomen.

**Figure 9:**
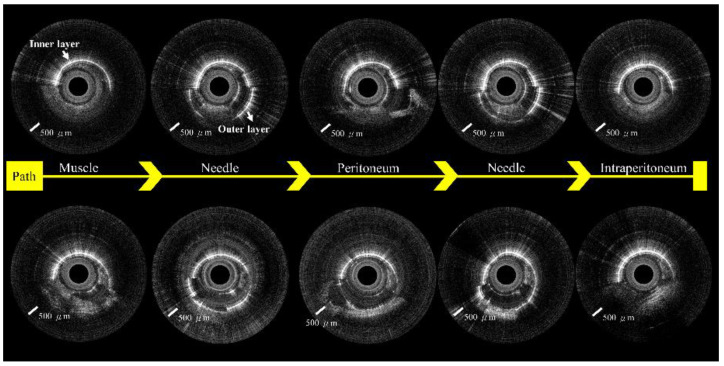
OCT images show the puncture path from the paramedian region of the belly.

We created a 5 cm full-thickness lateral abdominal wall incision for the visual confirmation of needle placement. The OCT images were significantly different before (Figure 7A) and after (Figure 7B) needle puncturing through the peritoneum. Figure 8 and Figure 9 show serial OCT images of the puncturing process (left to right) while the incorporated Veress needle was punctured through the midline and paramedian abdominal regions. The tissues that the needle passed through were, in order: muscle, peritoneum, and intraperitoneal cavity. The needle’s outer and inner layers were scanned simultaneously after the needle passed through the muscle layer (or before puncturing through the fascia layer) and before puncturing through the peritoneum.

**Figure 10:**
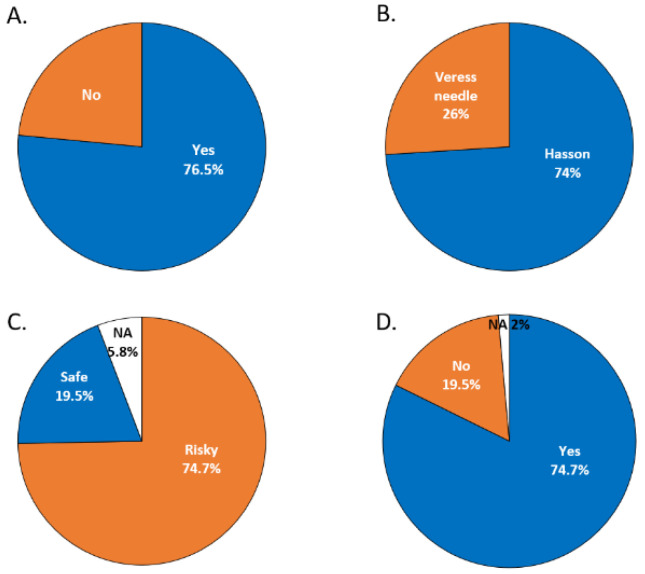
Peritoneal access survey among 150 surgeons. (**A**) Prior experience of Veress needle placement. (**B**) The current practice of peritoneal access. (**C**) Physicians’ opinions regarding the use of the Veress needle. (**D**) Will surgeons use Veress needle if the puncturing process could be visualized?

We surveyed 150 surgeons from various specialties, including general surgery, urology, gynecology, colorectal surgery, and pediatric surgery, regarding their practices of peritoneal access and their opinions on this issue. We found that among 150 surgeons, as high as 76.5% had the experience of using a Veress needle (Figure 10A). However, only 26% currently adopt the Veress needle, and 74% use the Hasson technique for peritoneal access (Figure 10B). The main reason the surgeons did not use the Veress needle was the concern of internal organ injury. Most of the surgeons (74.7%) considered that the insertion of the Veress needle was risky. Only 19.5% thought the procedure was safe (Figure 10C). If an assisted device could be offered to visualize the puncturing process, 74.7% of the surgeons would like to re-embrace the Veress needle for peritoneal access (Figure 10D). Based on this survey across different specialties, we could speculate the unmet need for the safe establishment of peritoneal access and thus urge the need to develop an assisted tool for access.

## 4. Discussion

Laparoscopy has become the standard of care in many operations and is widely used in many surgical specialties. Gaining access into the peritoneal cavity and creating a pneumoperitoneum is undoubtedly the first and most critical step in every laparoscopic procedure. Despite advancements in laparoscopic surgical tools, peritoneal access still carries the risks of internal organ and vascular injuries [3]. Therefore, there is a need to have an assisted technique to aid peritoneal puncture. In our experiments, the primary and secondary outcomes of the study were achieved. The success rate of peritoneal puncture into the peritoneal cavity was 97.5%. Our SSOCT system can provide a real-time display of the tomographic images from the incorporated Veress needle tip when the incorporated Veress needle punctures the abdomen’s distinct depth positions. The OCT image provided different tissue patterns during the extra-peritoneal tissue puncture, including the muscle, fat, and fascia. The extra-peritoneal fascia shows a V-shaped concave pattern similar to the peritoneum OCT image, but the peritoneum’s image intensity is stronger with lower image penetration than the extra-peritoneal fascia OCT image. Furthermore, the fascia and peritoneum’s V-shaped concave patterns significantly differed before the Veress needle punctured through them. The peritoneum’s V-shaped concave pattern was always more prominent than that of the fascia, owing to the greater elasticity of the peritoneum. We observed that the STD value of the OCT image of the peritoneum was higher than that of the extra-peritoneal fascia. A statistical difference indicated the differentiability of images between the peritoneum and extra-peritoneum (*p* < 0.005) in the STD value (Figure 6), indicating that the STD value had a high capacity for discriminating between the peritoneum and extra-peritoneal fascia. Thus, OCT imaging with the quantitative calculation of the STD value can further aid objective identification.

Some methods have been proposed to increase the safety of closed peritoneal access, including the open technique described by Hasson in 1971 [19]. The peritoneal cavity was entered under direct vision through a 1~1.5 cm sub-umbilical incision in the open technique. However, it can be more time-consuming, more challenging in patients with obesity, has an increased chance of gas leakage during the procedure, and still carries certain risks of vascular and bowel injuries [20]. Riek et al. designed a new insufflation needle with a transparent, conical tip to which an endoscope can be attached [14]. The progress through the abdominal wall could be observed under endoscopy. An updated version of the system consisted of a Veress needle with an integrated spring-activated protective shield and a fiberglass optic at the distal end of the protective shield [15]. The concept of the system was to apply endoscopy to visualize the puncturing progress. However, either a newly designed hollow insufflation needle with a diameter of 3.6 mm or a dedicated endoscopic system was needed in their equipment. Furthermore, the visualization quality and range of vision would be limited owing to the inherent characteristics of fiberoptic scope view. In our study, we used the OCT system as the visualization tool to offer a more extensive tomographic view than the fiberoptic scope view. Nevler et al. proposed an improved Veress needle for safer trocar insertion for closed laparoscopic access [21]. The new Veress needle includes a distal expandable portion that allows for the elevation of the abdominal wall and safe insertion of the first trocar over it. As opposed to our system, this new Veress needle aims to improve the safety of the first trocar insertion after the needle enters the peritoneal cavity. It is not intended to improve the safety of peritoneal puncture by the needle instead. A new mechanism has also been designed to improve the safety of peritoneal puncture with a Veress needle. Postema et al. designed a novel Veress needle preventing the puncturing acceleration of the tip of the Veress needle by decoupling the surgeon’s hand from the needle immediately after entering the abdomen [22]. They showed that it could reduce overshooting with a minimum of 50% in a standardized ex vivo setting on fresh porcine abdominal wall specimens.

Our preliminary experiments have some limitations. First, although the OCT images of the different layers of the porcine abdominal wall are specific, it seems to be user-dependent to immediately recognize the OCT images of different layers. Therefore, we have planned an automated recognition system using the STD value of the OCT signal to identify the images in the next stage of the experiments. Second, one of the key features of this system was to identify the V-shaped concave peritoneum before puncturing it. However, the peritoneum may be too thin or friable to deflect the Veress needle, and the needle may puncture it without too much resistance. Although we did not observe such a situation in our experience, more experiments will be conducted to verify this. Third, the characteristics of the peritoneum, e.g., elasticity, in different ages could be different, which might influence the performance of our system. For example, the elasticity of the pediatric peritoneum could result in difficulty in puncturing the peritoneum. However, one of the key features of our system was to identify the V-shaped concave peritoneum before puncturing it. The more elastic the peritoneum is (e.g., in younger children), the more pronounced the V-shaped concave pattern. Therefore, puncturing the pediatric peritoneum of different ages should not present a problem in our OCT system. Fourth, two attempts failed because of the significant separation of the abdominal wall layers while intentionally elevating the abdominal wall during the puncture. Separation of the abdominal wall layers will increase the difficulty in differentiating the fascial and peritoneal layers. Therefore, we recommend neutral or limited elevation of the abdominal wall while puncturing the abdominal wall. Finally, the cost of the OCT equipment is currently close to (or even lower than) a surface ultrasound system. However, because of the rapid pace of innovation in the OCT field, the cost and ease of using such modalities are improving rapidly.

In our experiments, the blind closed process of a peritoneal puncture could be translated to a visualized procedure by incorporating the conventional Veress needle with a fiber-probe SSOCT system. The safety of peritoneal access with a Veress needle would thus be increased.

## 5. Conclusions

In conclusion, by combining the OCT imaging tool with the Veress needle as an integrated system, the incorporated Veress needle could provide OCT images of different layers of the porcine abdominal wall, including the peritoneum, for easy access to the peritoneal cavity before gas insufflation. Using this OCT image-incorporated system, we made the blind closed technique of peritoneal access a visualized procedure that can improve the safety of peritoneal access. In our preliminary experiment, the success rate of peritoneal access was acceptable and further supported the feasibility of this concept. Therefore, the OCT system as an assisted tool could be one of the possible solutions that warrant further exploration. The study aims to improve the safety of closed techniques instead of replacing currently available techniques. Although this experiment is primarily a proof of concept, we believe that the idea deserves further investigation. In the future, the OCT system is expected to be used in laparoscopic access and to provide a safer, automatic identification system for minimally invasive procedures, which are an essential part of modern surgery.

## Figures and Tables

**Table 1:** SSOCT criteria for identifying muscle, fascia, peritoneum, and intraperitoneal tissue.

Tissue Type		Characteristic Features Visible on OCT Images
Extra-peritoneal tissue	Muscle	Non-uniform signal distribution, presence of layered structures
	Fascia	Speckled bright reflections indicating adipose tissue, less imaging penetration depth than muscle tissue, V-shaped concave pattern
Peritoneum		Strong and homogeneous laminar layer, deep V-shaped concave pattern
Intraperitoneal tissue		Appearance of numerous empty spaces, no reflective signal, or homogeneous laminar layer tissue

## Data Availability

The data that support the findings of this study are available from the corresponding author upon reasonable request.

## Supplementary Materials

The following supporting information can be downloaded at: https://www.mdpi.com/article/10.3390/biomedicines10020485/s1, Video S1: The puncturing process with animation and OCT images.
